# Neoadjuvant Chemotherapy in Locally Advanced Rectal Cancer

**DOI:** 10.3390/cancers12123611

**Published:** 2020-12-03

**Authors:** Federica Papaccio, Susana Roselló, Marisol Huerta, Valentina Gambardella, Noelia Tarazona, Tania Fleitas, Desamparados Roda, Andres Cervantes

**Affiliations:** 1 Department of Medical Oncology, Hospital Clínico Universitario de Valencia, INCLIVA Biomedical Research Institute, University of Valencia, Avda. Blasco Ibañez 17, 46010 Valencia, Spain; fpapaccio@unisa.it (F.P.); srosello@incliva.es (S.R.); mhuerta@incliva.es (M.H.); vgambardella@incliva.es (V.G.); noetalla@incliva.es (N.T.); tfleitas@incliva.es (T.F.); droda@incliva.es (D.R.); 2 Department of Medicine, Surgery and Dentistry “Scuola Medica Salernitana”, University of Salerno, Via S. Allende, 84081 Baronissi, Italy; 3 Centro de Investigacion Biomedica en Red (CIBERONC), Instituto de Salud Carlos III, 28029 Madrid, Spain

**Keywords:** high-risk locally advanced rectal cancer, total neoadjuvant treatment, watch and wait strategy

## Abstract

**Simple Summary:**

The outcome for patients with rectal cancer has significantly improved over the last thirty years. Previously, local relapses in the pelvis occurred in more than one third of all patients with apparently localized tumors. Total mesorectal excision was the first step to improve local control by reducing local relapses to less than 5%. Preoperative radiation, either short-course or long-course with concurrent administration of chemotherapy, was a second important step for reducing local relapses to a minimum, even in locally advanced tumors where a clean surgical resection was not possible or would not be curative. Magnetic resonance imaging is a very useful tool for locoregional staging and for properly selecting patients for preoperative treatment. Nowadays, we know that preoperative chemotherapy also provides better control of systemic relapses. Moreover, surgery can be avoided in 25% of patients and the watch and wait strategy is considered safe and curative.

**Abstract:**

Most clinical practice guidelines recommend a selective approach for rectal cancer after clinical staging. In low-risk patients, upfront surgery may be an appropriate option. However, in patients with MRI-defined high-risk features such as extramural vascular invasion, multiple nodal involvement or T4 and/or tumors close to or invading the mesorectal fascia, a more intensive preoperative approach is recommended, which may include neoadjuvant or preoperative chemotherapy. The potential benefits include better compliance than postoperative chemotherapy, a higher pathological complete remission rate, which facilitates a non-surgical approach, and earlier treatment of micrometastatic disease with improved disease-free survival compared to standard preoperative chemoradiation or short-course radiation. Two recently reported phase III randomized trials, RAPIDO and PRODIGE 23, show that adding neoadjuvant chemotherapy to either standard short-course radiation or standard long-course chemoradiation in locally advanced rectal cancer patients reduces the risk of metastasis and significantly prolongs disease-related treatment failure and disease-free survival. This review discusses these potentially practice-changing trials and how they may affect our current understanding of treating locally advanced rectal cancers.

## 1. Introduction

**Figure 1:**
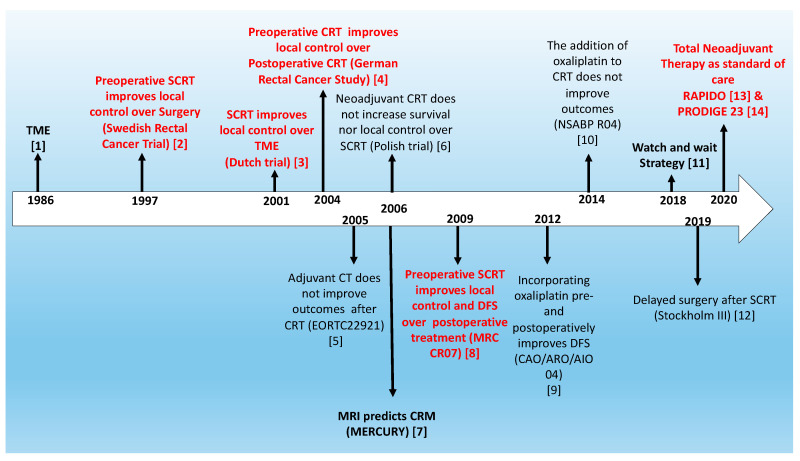
Evolution of rectal cancer treatment in the last decades. Randomized phase III trials that have improved outcomes are shown in red, and diagnostic tools and surgical/non-surgical approaches established with observational studies are in bold. TME: total mesorectal excision; SCRT: short-course radiotherapy; CRT: chemoradiotherapy; MRI: magnetic resonance imaging.

Rectal cancer accounts for around 35% of total colorectal cancer, and constitutes a major health concern. The outcome for patients with rectal cancer has significantly improved in the last thirty years. Previously, surgery was not standardized and local relapses in the pelvic area occurred in more than one third of all patients with apparently localized tumors. The introduction and global implementation of total mesorectal excision (TME) was the first step in improving local control by reducing local relapses to less than 5%. Preoperative radiation, which involves either a short course (SCRT) given for five days or a long course administered for five weeks with concurrent administration of chemotherapy, was a second important step in reducing local relapses to a minimum, even in locally advanced tumors, in which a clean surgical resection was not possible or would not have been curative. Locoregional staging with magnetic resonance imaging (MRI) is very useful for the proper selection of patients for preoperative treatment. Nowadays, we know that preoperative chemotherapy also provides better control of systemic relapses in those patients presenting high-risk features in whom metastatic progression is frequently observed. Moreover, surgery can be avoided in those patients that present a pathological complete response (pCR). The complete disappearance of all tumor cells in the surgical specimen has been observed in around 12% of patients after conventional preoperative chemoradiation (CRT) and up to 25% in patients receiving neoadjuvant chemotherapy as part of the total neoadjuvant treatment (TNT) strategy. These observations mean that surgery can be avoided in a higher proportion of cases, making the watch and wait approach more common, safe and curative. Figure 1 summarizes all of the relevant steps that have been taken with regard to the treatment of localized rectal cancer over the last three decades [1,2,3,4,5,6,7,8,9,10,11,12,13,14].

According to current clinical practice guidelines, localized rectal cancer treatment relies on accurate staging procedures, which is highly dependent on pelvic magnetic resonance imaging [15]. This allows the categorization of patients according to clinically defined risk categories. Each risk category may benefit from a specific type of treatment, for example, very early low grade cT1N0 can be treated with local excision, early stages are treated with upfront surgery, namely, total mesorectal excision [1] and low to intermediate risk patients benefit from preoperative treatment, which includes either short-course radiotherapy or conventional chemoradiotherapy based on fluoropyrimidines followed by TME [16]. 

**Figure 2:**
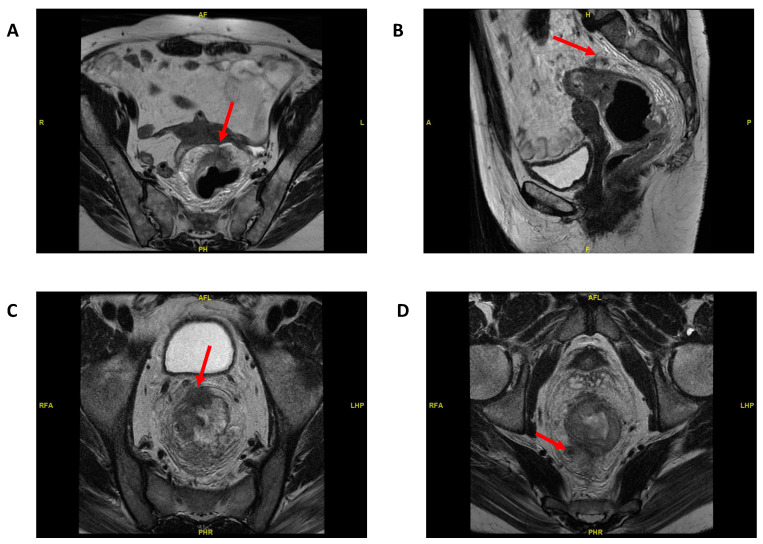
MRI from patients showing locally advanced rectal cancer with high-risk features. (**A**) Upper third rectal cancer with peritoneal reflection invasion (cT4a). (**B**) Same patient showing extra-mural vascular invasion. (**C**) Lower third rectal cancer in a male with invasion of the anterior part of the mesorectal fascia (cT3d) and multiple large size peritumoral lymph nodes (N2) (**D**).

Patients with locally advanced rectal cancer (LARC) may have at least one of the following MRI-defined high-risk features: T3 invading 5 mm or more, particularly those that involve or reach less than 1 mm of the mesorectal fascia, T4, extra-mural vascular invasion (EMVI), N2 and extra-mesorectal nodal involvement. Distant metastases are more frequently seen during patient follow up when these features are present. Figure 2 shows a staging pelvic MRI of a patient showing several high-risk features. In these patients, systemic relapses are significantly more frequent. Conventionally, they have been treated with preoperative CRT followed by surgery and in some cases, adjuvant chemotherapy according to the guidelines. However, the recently published RAPIDO and PRODIGE 23 trials have brought neoadjuvant chemotherapy to the fore as a new standard of care. 

Multidisciplinary team (MDT) discussion on appropriate initial treatment selection, including in some cases preoperative concurrent CRT or short-course radiotherapy (SCRT) has led to a dramatic improvement in locoregional control, with local relapses occurring in less than 5% of patients. Despite these achievements in local control, no major reductions in systemic relapse has been shown in randomized controlled trials, which precludes the determination of significant survival benefit. 

On the other hand, data derived from population-based registries tell a different story. The Norwegian rectal cancer registry analyzed the outcomes of more than 10,000 patients diagnosed with stage I–III rectal cancer from 1993 to 2010, and detected a statistically significant increase of almost 10% in relative five-year survival (71.2% in 1993–1997 vs. 80.6% in 2007–2010), and a decrease in distant recurrence rate of about 6% (26.0% vs. 20.2%, respectively) [17]. Moreover, in the International Cancer Benchmarking Partnership (ICBP), the Cancer Survival in High-Income Countries (SURVMARK-2) study, despite wide variations among different geographic areas, 5-year rectal cancer survival increased by more than 13% in Denmark, Ireland and UK, while other countries with better survival at study commencement saw a global increase of 8% [18]. Implementing selective neoadjuvant chemoradiation and proffering TME as the standard of care has led to improved global outcomes, including overall survival (OS), in population-based studies beyond those observed in academic randomized studies. These findings have recently been corroborated in a similar study by the Spanish Association of Surgeons, which conducted a retrospective analysis including more than 14,000 patients treated with curative intent between 2006 and 2017. They found a significant survival improvement over time in the whole population, and more intriguingly, a survival benefit that favored neoadjuvant chemoradiation, especially in patients with clinical stage III rectal cancer, and even more so in those with high-risk features including the involvement of the mesorectal fascia and N2 disease [19]. 

In the present review, we aim to outline the specific role of neoadjuvant chemotherapy in patients with locally advanced rectal cancer presenting with MRI-defined high-risk features. This approach was initially controversial, but in light of recently presented evidence, neoadjuvant chemotherapy before or after SCRT or concomitant CRT could become the current standard of care. We also discuss the potential value of neoadjuvant and/or total neoadjuvant treatment to increase pathological complete remission, and thus facilitate non-operative strategies in rectal cancer.

## 2. Total Neoadjuvant Treatment: A Strategy to Provide All Treatment Modalities 

No clear evidence exists for the efficacy of oxaliplatin-based adjuvant chemotherapy in rectal cancer as a way to improve systemic control [20,21], and concerns about treatment compliance in a postoperative setting are justified. On the other hand, intensified and neoadjuvant approaches combining different chemotherapy agents and different RT schedules have been explored in several clinical trials over the years [20]. Neoadjuvant chemotherapy has been shown to be feasible despite heterogeneity in the inclusion criteria for neoadjuvant chemotherapy studies, which include more selective MRI-driven approaches and others that use looser criteria. This kind of approach is sometimes termed total neoadjuvant therapy (TNT). The rationale for utilizing this approach include the early targeting of micro-metastatic disease, which is particularly relevant in high-risk LARC, it shows better tolerability and treatment compliance, and it results in an increased pathological complete response (pCR) rate, which can improve the R0 resection rate. On the other hand, by administering all chemotherapy before surgery, significantly more patients receive adequate systemic therapy compared to the conventional approach. Patients with surgical complications after conventional CRT, particularly those having an abdomino-perineal resection, may have delayed postsurgical recovery and may not receive timely postoperative chemotherapy. Moreover, TNT could have an impact on the complete response rate, and it has potential as an option in patients eligible for organ preservation strategies [22]. 

Until now, the bulk of data has come from phase 2 and retrospective cohort studies. In the Spanish GCR-3 phase II randomized trial, 108 patients selected by MRI staging were treated with preoperative CRT followed by surgery and adjuvant capecitabine and oxaliplatin (CAPOX) or four courses of neoadjuvant CAPOX followed by CRT and surgery. The primary endpoint was pCR rate, which varied little between treatment arms. No significant differences were detected in disease-free survival (DFS) or OS, even at longer follow-up. Besides these results, what particularly stands out for the purpose of this review is the data regarding compliance and toxicity. Indeed, compliance was higher in the induction arm (94% of patients received all four induction chemotherapy courses, while 76% received adjuvant treatment and only 57% received all planned cycles). Adverse events were significantly higher during adjuvant treatment than with induction chemotherapy [23,24]. These data on compliance have been confirmed in the phase 3 RAPIDO trial’s preliminary results, showing significantly higher compliance in the neoadjuvant chemotherapy arm than when chemotherapy was given postoperatively (84% vs. 58%) [25]. Interestingly, tolerance was also improved after triplet combination chemotherapy in the recently presented PRODIGE 23 trial [26].

Furthermore, although retrospective, some relevant conclusions can be drawn from the Memorial Sloan Kettering (MSK) experiment [27]. It reports data on approximately 300 patients with LARC treated with TNT compared to a similar number of patients treated with CRT. Higher exposure to chemotherapy was detected in the TNT cohort patients in terms of total dose, dose reductions and number of planned courses. The complete response rate, including both pCR and clinical complete response (cCR) in patients not subjected to surgery, was 36% in the TNT cohort compared with 21% in the CRT cohort. A systematic review evaluated the oncological outcomes of induction chemotherapy before or after preoperative CRT in a total of 648 patients from ten studies, with the majority being phase 2. A DFS and OS rate of more than 60% and 70%, respectively, were reported [28]. 

A phase 2 randomized study by a German group (the CAO/ARO/AIO-12) evaluated the TNT approach as either induction FOLFOX followed by CRT with 5-fluorouracil (5FU) plus oxaliplatin followed by TME, or the inverse strategy know as consolidation (CRT with 5FU and oxaliplatin followed by consolidation FOLFOX followed by TME). The pCR rate was higher with the second approach (25% vs. 17%) [29]. Thus, administering chemotherapy in the window between radiation completion and surgery was shown to be the most beneficial strategy. Moreover, the PAN-EX study, which selected only patients with high-risk features and pooled data from two phase 2 trials, highlighted the feasibility and favorable outcomes of neoadjuvant chemotherapy in high-risk LARC patients [30].

Until now the only phase 3 results that have reported on a TNT approach are derived from the Polish II study. In this trial, patients with cT3c/cT4 tumors were randomized to receive SCRT followed by three courses of FOLFOX4 or fluoropyrimidine-based CRT concomitant with weekly oxaliplatin. No significant differences were detected between treatment arms in terms of survival [31,32]. Nevertheless, this study has some weaknesses that limit its conclusions, such as the non-use of MRI for patient selection, the primary endpoint (R0 resection rate) and the 2012 protocol amendment, which allowed oxaliplatin withdrawal at the investigators’ discretion. Based on these data, this treatment strategy remains controversial and has been addressed in guidelines as an option for discussion.

Overtreatment is definitely a potential drawback of the expanded use of TNT. Some patients with LARC may show very good responses and excellent local control with conventional CRT. However, systemic relapses are very prevalent in patients that present MRI-defined high-risk features. Overtreatment could be substantially avoided by limiting TNT to those individuals that are selected by a detailed revision of the staging MRI at weekly multidisciplinary team meetings. In patients with less extensive tumors with very limited mesorectal invasion and located far from the mesorectal fascia and in the absence of lateral or extramesorectal nodes with no extramural vascular invasion, conventional CRT could be recommended with satisfactory outcomes, thus avoiding the addition of further toxicities derived from neoadjuvant chemotherapy.

## 3. Total Neoadjuvant Treatment as a New Standard of Care for LARC

**Figure 3:**
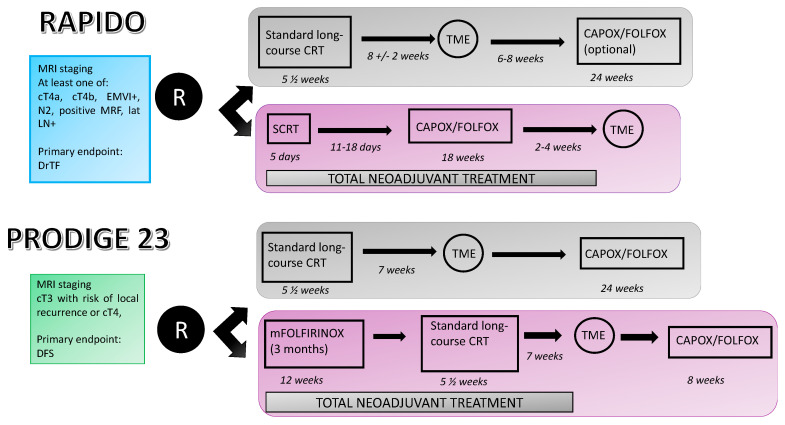
Comparison of RAPIDO and PRODIGE 23 trial designs. MRI: magnetic resonance imaging; EMVI: extra-mural vascular invasion; lat LN+: lateral lymph nodes involved; DrTF: disease-related treatment failure; MRF: mesorectal fascia; DFS: disease-free survival; CRT: chemoradiotherapy; TME: total mesorectal excision; SCRT: short-course radiotherapy.

The use of neoadjuvant chemotherapy in the treatment of LARC was recently revolutionized at the ASCO 2020 virtual meeting when the results of two pivotal randomized phase 3 trials, the RAPIDO [13] and the PRODIGE 23 [14] trials were presented. Both studies deal with TNT in LARC patients and establish a new standard of care. Figure 3 shows the design of these two trials. However, they have several differences, which are further discussed below and are described in Table 1 and Table 2.

An important phase 3 study, the Stockholm III trial, demonstrated that surgery can be safely delayed after SCRT for up to 12 weeks, and that this approach leads to an increased pCR rate without affecting postoperative complications [12,33]. Other studies have also examined the possibility of exploiting this window to administer systemic treatment, and ultimately, to move the regimen administered in the adjuvant setting to the preoperative one [34,35]. This approach was also used in the RAPIDO trial.

Interestingly, the Dutch M1 trial demonstrated that in patients with stage IV rectal cancer, administering SCRT on the primary tumor followed by FOLFOX-bevacizumab chemotherapy and then surgery after 6–8 weeks resulted in downstaging the primary tumor in 47% of patients with a 26% pCR rate in the primary tumor [36], and almost one third of patients were alive after a median follow up of more than 8 years [37].

The patient population in the RAPIDO trial consisted of those with MRI-defined high-risk locally advanced disease including cT4a/b, extramural vascular invasion, cN2, involved mesorectal fascia or enlarged lateral lymph nodes considered to be infiltrated. It is important to note that pelvic MRI was mandatory. Patients randomized to the experimental arm received SCRT followed by six cycles of CAPOX or nine cycles of FOLFOX followed by TME. Patients in the control arm received standard CRT followed by TME 8–10 weeks after CRT completion. Adjuvant chemotherapy was allowed depending on the investigator’s criteria.

When the trial commenced, the primary endpoint was DFS but in 2016 this was amended to time to disease-related treatment failure (tDrTF), which included locoregional failure, distant metastasis, new primary colorectal tumor or treatment-related death. This parameter may be a more appropriate endpoint for trials including neoadjuvant treatment. In fact, when surgery is planned with such a long delay, as in the experimental arm in the RAPIDO trial, conventional DFS status is only reached after surgery, potentially missing patients that are progressing during neoadjuvant treatment. Later, in 2017, a change was also made to the statistical hypothesis due to the low event rate at the second planned interim analysis. Between 2011 and 2016, 920 patients were included. The study met its primary endpoint, showing a statistically significant benefit in tDrTF at 3 years with an HR of 0.75. The 3-year DrTF rate was lower in the experimental arm (23.7% vs. 30.4%), as was the 3-year distant metastasis rate (20% vs. 26.8%). The local relapse rate difference was not statistically significant between the two arms: 8.3% in the experimental vs. 6% in the standard arm (*p* = 0.12). This occurred despite the higher quality of mesorectal surgery in the standard arm compared to the experimental arm (85% vs. 78% intact mesorectal plane, respectively, *p* = 0.032) [25]. Also, 92.2% of patients allocated to the experimental group underwent curative surgery vs. 88.9% in the control arm (*p* = 0.086), an important finding considering the population of the trial, which had a high local disease burden. 

As regards pathological response, it is noteworthy that 28.4% of patients in the experimental group achieved pCR compared to 14.3% in the control arm. As expected, higher toxicity was detected in the experimental arm, due to the intensive treatment. During neoadjuvant treatment, grade ≥3 adverse events occurred in 48% of patients in the TNT arm, and in only 25% of the standard CRT arm. Diarrhea was the most prevalent toxicity seen during neoadjuvant treatment in both groups. During postoperative chemotherapy in the standard arm, 35% of patients had at least a grade ≥3 adverse event. However, there were more serious adverse events in the control than experimental arm (42% vs. 39%). Moreover, a high compliance to systemic treatment was achieved with the experimental schedule of SCRT followed by preoperative systemic chemotherapy with FOLFOX or XELOX. Although considerable preoperative toxicity was reported compared to CRT, no differences were found in the details of the surgical procedures, the proportion of patients undergoing surgery and the rate or severity of postoperative complications. At the time of reporting, the 3-year OS rate was not significantly different between the two arms, but the number of events is too low (less than 18% of patients deceased in both arms) to assess OS as a secondary endpoint at this time. 

The PRODIGE 23 trial investigated an even more intensified regimen, which consisted of triplet mFOLFIRINOX chemotherapy (5-fluorouracil, irinotecan and oxaliplatin) added to standard CRT. The target population was patients with either stage II or III rectal cancer, thus patients without MRI high-risk features were also included. As in RAPIDO, pelvic MRI was mandatory in this study. The standard arm consisted of preoperative CRT followed by surgery and then adjuvant chemotherapy. The experimental arm consisted of six courses of mFOLFIRINOX followed by standard CRT, surgery and adjuvant chemotherapy. The choice of adjuvant treatment was mFOLFOX or capecitabine and was left to the center’s discretion. The primary endpoint of this trial was 3-yr DFS. 

Between 2012 and 2017, 461 patients were included in this study, which also met its primary endpoint, and showed an increase in 3-year DFS in favor of the experimental arm: 75.7% vs. 68.5% (HR 0.69). Three-year metastasis-free survival was higher in the triplet chemotherapy group (78.8% vs. 71.7%). The experimental arm almost tripled the rate of pCR (27.5% vs. 11.7%) compared to standard CRT. Survival data are not yet available. Overall, the authors report that despite the complexity of the treatment it was well tolerated, and patients were able to complete it. Indeed, authors did not observe significant differences in quality of life scores between the treatment arms [26]. 

Both the RAPIDO and PRODIGE 23 trials demonstrate a clinically relevant and statistically significant decrease in relapses as well as an increase in pCR. Although important, the quantitative effect observed is moderate. Nonetheless, if the ESMO Magnitude of the Clinical Benefit Scale for potentially curative therapies was applied both trials would score as A, because they show an improvement in DFS or DrTF alone (primary endpoint) with the lower limit at the 95% confidence interval HR below 0.65 without mature survival data [38]. However, the intensity of both neoadjuvant chemotherapy schedules would certainly limit their use in elderly patients. In fact, the median age of participants was 62 years in both studies and only 11% of the patients in the PRODIGE 23 trial were over 70.

Although the two trials have several similarities, they have key differences that shape their different responses to clinical questions (Table 1). It is evident that RAPIDO was far more selective in its inclusion criteria, and around 40% of patients received no adjuvant treatment, as per hospital policy, while in the PRODIGE 23 trial it was part of the treatment protocol. Therefore it could be argued that a subset of patients in PRODIGE might have been overtreated, while it is unclear from the study whether there is a subpopulation that really benefits from intensified treatment. In contrast, due to its straightforward and stringent MRI-defined high-risk selection criteria, the RAPIDO trial addresses a target population with very poor prognosis for whom current treatments might be insufficient. 

## 4. Is There a Role for Neoadjuvant CT in Organ Preservation Strategies? 

One compelling medical question that needs to be addressed concerns patients who achieve cCR after preoperative treatment: is it possible to spare them surgery? Large-scale registries of individual patient data are encouraging and report an 8% distant metastasis rate, 85% 5-year OS and 94% 5-year disease-specific survival [11]. To date, there is no randomized controlled evidence regarding a watch and wait (WW) strategy in this disease setting, probably because of the lack of a consensual definition of cCR. However, current evidence indicates that by increasing pCR rates, neoadjuvant chemotherapy can also increase the proportion of candidates eligible for a nonsurgical approach. Indeed, a retrospective analysis on the outcomes of patients achieving cCR has shown the feasibility of this approach, despite the small number of patients [39].

In a study published in 2015, administering mFOLFOX6 after CRT increased the proportion of patients achieving pCR. Interestingly, the more courses that were administered, the higher the pCR rate found: 18% with no added treatment, 25% with 2, 30% with 4 and 38% with 6 courses [40]. Some encouraging data emerged from the above-cited MSK cohort [27]. In the CRT group (*n* = 320) 19 out of 24 patients not subject to surgery had cCR (approximately 6% of the total cohort). In the TNT group (*n* = 410), 67 out of 73 patients who did not undergo surgery had cCR (approximately 16% of the total cohort). These data suggest that use of TNT can spare a higher number of patients from surgery.

**Figure 4:**
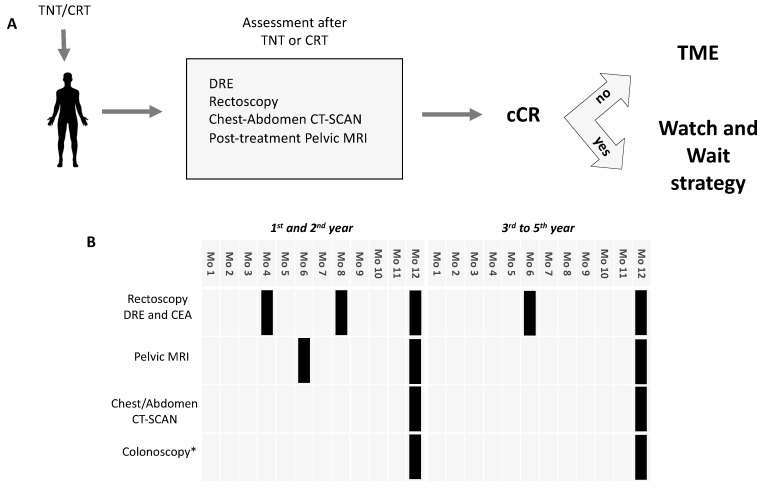
(**A**) Flow chart for assessment, selection and implementation of a watch and wait strategy. (**B**) Follow-up time table during watch and wait strategy. TNT: total neoadjuvant treatment; CRT: chemoradiotherapy; DRE: digital rectal examination; MRI: magnetic resonance imaging; cCR: clinical complete response; Mo: months. * colonoscopy is to be performed one year after diagnosis and thereafter as per guidelines.

The recently presented OPRA trial sheds some light on this field and gives some possible applications of neoadjuvant chemotherapy [41]. This phase 2 study randomized stage II–III rectal cancer patients to receive four months of induction FOLFOX or CAPOX given before CRT or consolidation FOLFOX or CAPOX given after CRT. At 8–12 weeks after completion of neoadjuvant treatment patients were restaged. Those with cCR (or near) were offered a WW strategy, while the others had TME. The primary aim was to compare the 3-year DFS of patients treated with neoadjuvant chemotherapy and WW or TME with historical controls. A total of 324 patients were included. Overall, there was no difference in 3-year DFS in the induction and consolidation group compared to historical controls. Moreover, the order of treatment (induction or consolidation) did not seem to affect survival outcomes, while the consolidation arm seems to be associated to a higher rate of WW compared to induction arm. The authors concluded that this strategy could be an option for patients achieving cCR after neoadjuvant chemotherapy. Figure 4 shows the clinical flow of patients with rectal cancer with clinical complete remission who avoid surgery within the watch and wait strategy.

Randomized studies clearly show the difficulties of implementing a WW strategy. However, it is likely that more patients will request evaluation for a nonoperative approach in the near future. Neoadjuvant chemotherapy will induce more pCRs and lead to a greater number of nonsurgical procedures, which is an acceptable trade-off that can be monitored by international registries to provide useful information on how the situation may be evolving.

## 5. Conclusions

Neoadjuvant chemotherapy with CAPOX or FOLFOX added after short-course radiation and delayed surgery or upfront mFOLFIRINOX before long-course chemoradiation followed by surgery are two validated options for treating LARC with MRI-defined high-risk features. Both approaches have been validated in randomized phase III studies that have shown a clinically relevant and statistically significant reduction in disease-related treatment failure or improved disease-free survival. Moreover, fewer metastatic relapses and more pCRs were observed. In summary, total neoadjuvant treatment has been recognized as a new standard of care. Following the 2004 publication by Sauer et al. in the New England Journal of Medicine [4], which confirmed the value of preoperative chemoradiation in reducing local relapses vs. the conventional postoperative approach, no trial has been able to demonstrate its effect on reducing the risk of systemic relapses or provide the rationale for it to become a new standard of care. This has now been made possible thanks to the commitment of research groups and the enthusiasm of clinical investigators who believe in clinical research as the only way to provide a better future for rectal cancer patients.

## Figures and Tables

**Table 1:** Comparison of the patient characteristics in the RAPIDO [13] and PRODIGE 23 [14] trials.

Patient Characteristics	RAPIDO (TNT vs. CRT)	PRODIGE 23 (TNT vs. CRT)
Median age	61 yrs vs. 61 yrs	61 yrs vs. 62 yrs
Patients enrolled	462 vs. 450	231 vs. 230
cT4 (%)	30.4% vs. 31.8%	17.8% vs. 15.6%
cN2 (%)	68% vs. 68%	Not stated
EMVI+ (%)	32% vs. 28%	Not stated
MRF involved	62% vs. 60%	26% vs. 27.7%

CRT: chemoradiotherapy; TNT: total neoadjuvant chemotherapy; EMVI: extramural vascular invasion; MRF: mesorectal fascia; yrs: years.

**Table 2:** Comparison of the outcomes of the RAPIDO [13] and PRODIGE 23 [14] trials.

Outcomes	RAPIDO	PRODIGE 23
	(TNT vs. CRT)	(TNT vs. CRT)
Median FU	4.6 yrs	3.8 yrs
Primary endpoint	3-yrs DrTF	3-yrs DFS
	23.7% vs. 30.4% (HR 0.75 [95% CI 0.60–0.96]; *p* = 0.019)	75.7% vs. 68.5% (HR 0.69 95% [CI 0.49–0.97]; *p* = 0.034)
3-year MFS	80% vs. 73.2%	78.8% vs. 71.7%
pCR rate	28.4% vs. 14.3%	27.5% vs. 11.7%
Local relapse	8.7% vs. 5.4%	4.8% vs. 7%
3-year OS	89.1% vs. 88.8%	90.8% vs. 87.7%

FU: follow up; CRT: chemoradiotherapy; DrTF: disease-related treatment failure; DFS: disease-free survival; TNT: total neoadjuvant chemotherapy; pCR: pathological complete response; OS: overall survival; yrs: years.
